# Analysis of spatial heterogeneity in Xi'an's urban heat island effect using multi-source data fusion

**DOI:** 10.1371/journal.pone.0332885

**Published:** 2025-10-17

**Authors:** Yuan Meng, Qian Luo, Boyu Bai, Yonghao Li, Jialin Lu, Juan Ren

**Affiliations:** 1 School of Architecture, Chang’an University, Xi’an, Shaanxi, China; 2 School of Architecture, Southeast University, Nanjing, Jiangsu, China; 3 School of Architecture, South China University of Technology, Guangzhou, Guangdong, China; 4 School of Architecture, Xi’an University of Architecture and Technology, Xi’an, Shaanxi, China; The Chinese University of Hong Kong, HONG KONG

## Abstract

In the context of global climate change, this study aims to investigate the spatial heterogeneity and driving mechanisms of the urban heat island (UHI) effect within Xi’an’s second ring road area. We constructed a novel multi-source data fusion framework that integrates high-resolution remote sensing imagery, detailed building spatial data, and semantic indicators from street view imagery. Based on this framework, we extracted seven key environmental features and land surface temperature (LST) data. We employed Multi-scale Geographically Weighted Regression (MGWR) and machine learning models, including Random Forest, XGBoost, and Gradient Boosted Regression, to analyze both nonlinear interactions and spatially localized variations influencing UHI intensity. The results indicate that building density (BD), green view index (GVI), and road density (RD) are the dominant factors affecting LST, showing significant spatial heterogeneity. BD has the highest global importance with a SHAP value of 0.665 in the XGBoost model and shows positive effects on LST, especially in high-density areas. GVI exhibits stable negative correlations with LST, highlighting its cooling potential in medium- to high-density zones. MGWR regression coefficients for BD and GVI range from −0.66 to 1.38 and −0.53 to 0.33, respectively, revealing substantial local variation. Our analysis reveals the necessity of spatially differentiated climate adaptation strategies, and confirms the effectiveness of fine-grained environmental indicators in representing UHI formation mechanisms. The proposed multi-source data fusion and integrated MGWR-machine learning framework offers refined methodological tools and practical insights for enhancing urban thermal resilience and developing targeted microclimate regulation policies.

## 1 Background

The urban heat island (UHI) has become a pervasive and increasingly serious environmental problem in the process of global urbanization. The phenomenon, manifested as higher urban temperatures compared to the surrounding countryside, is mainly caused by complex factors such as topographic structure, increased impermeable surfaces and high population density [1]. Subsurface materials in urban environments, such as concrete [2] and asphalt [3], possess low albedo and high heat capacity, which causes them to retain heat for extended periods. And this, together with the heat released by human activities, leads to the formation of high-temperature centres in the centre of the city, which diminish as one moves the surrounding areas [4].

Xi’an, as a high-density inland city, exhibits complex UHI characteristics that call for refined and integrated analytical approaches. To address this, we propose a multi-source data fusion framework integrating remote sensing, building geometry, and street view semantic segmentation, enabling a more comprehensive understanding of UHI dynamics. The main contributions of this study are threefold: (1) we develop a high-precision data fusion framework aligning 3D morphological features and LST distribution; (2) we introduce an integrated MGWR–machine learning approach to capture nonlinear and spatially varying relationships; and (3) we conduct an in-depth spatial analysis of Xi’an’s urban core using interpretable AI models.

The remainder of the paper is structured as follows: Section 2 reviews the relevant literature and theoretical foundation; Section 3 describes the data and methods; Section 4 presents the results and analysis; Section 5 discusses implications and limitations; and Section 6 concludes the study.

## 2 Review

The research methods on the UHI effect can be divided into four main categories: theoretical analysis, observation methods, simulation methods, and integration methods [5]. Early studies mainly focused on theoretical analysis and observation methods, such as Oke [6] ‘s proposal of the energetic basis of UHI in 1982. With the advancement of technology, simulation methods, and integration methods have gradually become the mainstream of research. Table 1 combines some of the relevant literature on heat island research and analyzes the methods used in different studies.

**Table 1:** Literature combing related to heat island research.

Articles	Method	Routes	Features
Stewart et al., 2012 [7]	law of conservation of energy	LCZ classification, Energy balance equation	Method standardization
Arnfield et al., 2003 [8]	Field observation	Field observation, Statistical analysis	Actual situation reflection.
Voogt et al., 2003 [9]	thermal imaging remote sensing observation	Relationship model with land cover and building density	Large-scale map of heat island phenomena
Oke et al., 1982 [6]	Energy balance model	Energy balance equation, Parameterization	Basic theory, Fast calculation speed
Zhao et al., 2018 [10]	Numerical simulation Energy balance	Urban-climate modeling, Scenario analysis	Reveals UHI-heatwave synergy
Cermak et al., 1984 [11]	Small-scale model method	Small-scale urban model, Parameterization	High-resolution information
Martilli et al., 2002 [12]	CFD mesoscale model	Parametric scheme of urban surface exchange	Mesoscale meteorological research
Tominaga et al., 2008 [13]	CFD microscale model	Simulation analysis and optimization design	High-resolution information
Mekhloufi et al., 2025 [14]	Remote sensing, Open-source GIS tools	Free satellite data analysis, Urban green mapping	Low-cost, Flexible toolchain
Shahfahad et al., 2024 [15]	Remote sensing time-series	Temperature range mapping, Extreme heat detection	Identifies heat risk zones
Wang, et al., 2021 [16]	Spatial analysis	Building height effect on urban heat	Quantifies vertical form effects
Skamarock et al., 2005 [17]	Integration of microscale and mesoscale models,	Coupling framework, Bidirectional data transfer	Comprehensive high resolution
Wei et al., 2023 [18]	Street view images combined with land cover data	Machine learning, Regression models Predict LST accurately	Extensive range, High resolution
Chiang et al., 2023 [19]	Street view images, Deep learning	Sky view factor, Greenery mapping	Extracts urban form indicators
Oliveira et al., 2020 [20]	Based on geographic information and LZC classify-cation	Merge and transform data, LZC classification	High accuracy and operability of UHI analysis
Huang et al., 2025 [21]	Street view images, Satellite integration	Urban green detection, LCZ analysis	Integrates multi-source imagery
Wen et al., 2024 [22]	Comparative spatial analysis	Urban form effects on LST	City-specific findings
Liu et al., 2019 [23]	remote sensing technology combined with CFD simulation analysis	Remote sensing technology, CFD simulation, Machine learning	Overall assessment, Urban scale assessment
Melika et al, 2024 [24]	InSAR and GNSS multi-source data fusion	Integrate data, LCZ classification, Machine learning	Multi-source data advantage synthesis
Qiquan et al., 2024 [25]	remote sensing data, ground observation data, and DEA method	Multi-source data fusion, Cross-validation, Actual data observation	Improve the accuracy of UHI intensity estimation

However, theoretical analysis methods, while providing a macroscopic understanding of the heat island phenomenon [7], have limitations in accurately predicting the heat island effect situation in specific cities [5].

Observational methods can provide direct observation data, but the data coverage is limitedby meteorological conditions and observation networks [5]. For example, Arnfield et al [8] studied the factors associated with the heat island effect through field observations. However, due to the limited data coverage, it is difficult to reflect the UHI effect [5] fully. Voogt et al [9], on the other hand, investigated the influence of different land cover types on the heat island effect by using thermal imaging remote sensing, but there are challenges in obtaining the temperature information of vertical structures such as street canyons [5]. Although these observational methods are effective in specific regions and environments, their application at urban scales and time scales may be limited.

With the development of technology, simulation methods have gradually become the mainstream of research. For example, the energy balance model proposed by Oke et al [6] has been widely used in large-scale energy consumption studies, but its accuracy still needs to be improved [5]. Martilli et al [12] used a CFD mesoscale model to predict the heat island phenomenon, but the consumption of computational resources is large, and the model parameterization is complicated [5]. Microscale CFD models are more prominent in providing higher resolution flow field information [13], but their applicability is limited, especially when simulating large-scale cities [5]. These numerical simulation approaches have proven effective in various built environment applications, from indoor ventilation strategies in healthcare facilities to urban-scale thermal analysis [26].

In order to overcome the limitations of single simulation methods, integrated methods that combine multiple data sources and technological tools have emerged [5]. Mekhloufi et al. provide a valuable, up‑to‑date contribution by demonstrating how free, open‑source satellite data and tools can be effectively applied to assess urban green space dynamics and their influence on surface temperature patterns over nearly two decades in Algiers, North Africa. We adopt a proactive environmental strategy perspective to guide the selection and interpretation of environmental variables, aligning with the theoretical constructs found in sustainability and competitiveness research [14,27,28] is conceptualized as a reflection of compact development pressure. These integrated methods can combine data from different scales and sources to improve the accuracy and reliability of heat island effect prediction. For example, Skamarock et al [17] combined a microscale CFD model with a mesoscale meteorological model to achieve better results, and Wei et al [18] improved the prediction of heat island effect by combining street view images and land cover data.

The development of integrated methods has also facilitated research related to the UHI effect in terms of spatial heterogeneity. Current research on the spatial heterogeneity of UHI focuses on the multi-dimensional driving mechanism and regional differentiation law. In terms of driving factors, some scholars focus on the synergistic effect of multiple factors, such as the spatial coupling of two-dimensional morphological indicators and three-dimensional architectural structures, which reveals the nonlinear superposition of elements [29]; some studies also focus on a single dominant factor, such as the morphology of the water body or the degree of landscape fragmentation, to analyse its spatial and temporal differentiation and elucidate the local regulatory mechanism [30,31]. Prior studies have quantitatively demonstrated the significant impact of building height on land surface temperature (LST). For instance, Wang and Xu revealed a strong negative logarithmic relationship between building height and LST within the 0–66 m range across six Chinese megacities. They also confirmed that vertical urban expansion, represented by increased building height, plays a crucial role in mitigating LST, highlighting the cooling potential of high-rise development in compact urban area [16]. In terms of regional differences, intra-city studies focus on comparing the intensity of heat islands in the central city with that in the expansion areas, and on the temperature heterogeneity of LCZs or functional zones [32]; while inter-city studies reveal the geographic pattern of spatial differentiation of heat islands by comparing the macro-variables of climatic backgrounds, topographic features, or urban morphology [33,34]. Methodologically, GWR/MGWR and geoprobes are widely used to quantify the local effects of neighbourhood-scale morphology indicators [35]. Some studies have employed the Geographical Spatiotemporal Weighted Regression (GTWR) model, aiming to simultaneously capture the heterogeneity of driving factors in both spatial and temporal dimensions [36]. This model demonstrates strong capabilities in revealing the spatiotemporal dynamic evolution of driving factors. However, the MGWR model, by independently optimizing the specific spatial bandwidth for each variable, can provide higher accuracy in capturing pure spatial non-stationarity. Recent studies have revealed complex UHI–HW interactions in cities like Delhi and the United States, showing a significant rise in summer temperatures from 1991–2018 [10,15]. Spatial heterogeneity under HWs remains underexplored [37], despite evidence that land cover types affect UHI responses. Large-scale research also highlights serious public health risks [38]. These findings call for detailed investigation into spatial drivers such as BD, GVI, and radiation.

Although significant progress has been made in the current research on spatial heterogeneity of UHI, there are still two aspects of research gaps:

Firstly, in terms of the research scale and objects, the existing works mostly focus on macroscopic comparisons between cities or comparisons of discrete functional areas within a city, such as different local climate zones (LCZ) or land use types. Although they can reflect regional differences, they are difficult to capture the fine and continuous spatial gradient changes of the thermal environment in high-density continuous built-up areas. Driving factors such as building form and green space layout often play a role at the microscale, and their local effects are easily smoothed out in fragmented or large-scale analyses. Systematic research on the gradient characteristics and driving mechanisms of the thermal environment in continuous built-up areas of high-density inland cities (such as Xi’an, etc.) is still relatively lacking.

Secondly, at the level of data integration and analysis methods, although multi-source data fusion has improved the model accuracy, the existing integration methods still generally face technical challenges such as high data quality requirements, inconsistent spatial matching and scale of heterogeneous data, which can easily lead to analysis deviations. At the same time, the research on integrating multi-dimensional data such as macro remote sensing, three-dimensional building form and street scene images, and conducting small-scale detailed modeling and multi-factor nonlinear interaction analysis based on this is still insufficient. In particular, systematic research combining “high-density continuous spatial units” with “multi-source data collaborative analysis” is still not common among most.

In response to the aforementioned gaps, under the premise of selecting Xi’an’s high-density urban area as the research subject, we innovatively integrates high-resolution street view images, refined building spatial data, and remotely sensed LST data, from which the independent and target variable indicator data are extracted, to construct a multi-scale fusion integrated analysis framework, aiming to make up for the deficiencies of the existing methods in the analysis of vertical thermal environments and the modeling of spatial heterogeneity, to explore the spatial heterogeneity of the UHI effect and its influencing factors in depth: This framework precisely coordinated and spatially aligned remote sensing images, building space data, and street scene images, forming a unified analysis unit that can simultaneously reflect the macroscopic temperature field, three-dimensional vertical physical form, and street-level environmental characteristics. This laid a reliable data foundation for detailed research. At the same time, we developed a hybrid analysis method that combines MGWR with machine learning. This method fully leverages the advantages of each: The MGWR model is good at capturing how multiple driving factors and surface temperature change with spatial geographical location from a global perspective; while the machine learning mode can precisely depict the influence of each driving factor and its complex nonlinear interaction with the urban heat island phenomenon, thereby revealing its local effects. The combination of these two methods achieves complementary and unified “global prediction” and “local explanation” of the UHI driving mechanism, providing a stronger scientific basis for formulating locally adapted heat mitigation strategies.

## 3 Research methods

Xi’an is located in central China (coordinates: 34°16′N, 108°56′E), serving as the capital of Shaanxi Province. The city lies within a warm temperate semi-humid continental monsoon climate zone, with an average annual temperature of 13.3°C and average precipitation of 650 mm. According to the 2020 census, the population within the Second Ring Road—the core urban area focused in this study—was over 4.3 million. The area includes a mix of historical neighbourhoods, high-density commercial zones, and newly developed residential communities. The city’s compact core area within the Second Ring Road provides a typical example of a high-density urban environment in western China. Moreover, the availability of multi-source open-access data—such as satellite imagery, building footprints, and street view images—makes it suitable for integrated urban heat analysis. This study aims to explore the spatial heterogeneity of the UHI effect within the second ring road of Xi’an and its main influencing factors through multi-source data fusion and advanced model construction. To this end, this paper adopts a three-level research process of data collection, pre-processing, and analytical modeling as follows.

### 3.1 Research design

This study adopts an integrated spatial analytical design that combines geospatial modeling with machine learning techniques to explore the UHI effect. Xi’an’s urban core within the second ring road was selected as the study area due to its typical high-density development and significant thermal disparities. The objective is to understand how urban form and environmental features jointly influence land surface temperature (LST) patterns by leveraging a multi-source data fusion strategy.

The Landsat-9 image acquired on 23 May 2024 was selected because late-spring daytime conditions (May–June) consistently produce strong urban-heat-island contrast in Xi’an, maximizing the spatial signal for our analysis. Only scenes with 0% cloud cover across the study area were considered to eliminate radiometric artefacts caused by cloud contamination or atmospheric-correction uncertainty.

### 3.2 Measures

Data pre-processing work mainly includes the extraction of independent variables and target variables, data integration, and spatial matching; the specific steps are as follows:

(1) **Sample point construction and image processing**

Vector road network data was obtained from OpenStreetMap (https://www.openstreetmap.org/) and processed in ArcGIS Pro using buffer analysis. The road data were simplified to single-line representations through rasterization and vectorization. Based on this network, 3024 sampling points were placed at 25-meter intervals throughout Xi’an’s second ring road. At each point, street view images were acquired in four directions (0°, 90°, 180°, and 270°) using the Baidu Map API, and were then stitched into panoramic images using a Python script [39]. The selected indicators—GVI, SVI, BD, RD, etc.—are closely related to urban morphological and perceptual variables affecting thermal performance. GVI and SVI reflect vegetation and sky openness from the pedestrian perspective, while BD and RD capture built-up intensity and traffic exposure. These indices have been validated in prior UHI studies for their interpretability and spatial relevance [19,21].

Semantic segmentation was performed using the ResNet50dilated + PPM_ deepsup model trained on the ADE20K dataset, which includes 20,210 images for training, 2,000 images for validation, and 3,000 images for testing. The ADE20K dataset provides diverse and complex urban scenes, making it well-suited for training models in street-level semantic analysis.

In this study, the ResNet50dilated + PPM_deepsup model achieved strong performance on the test set, with a Mean Intersection over Union (IoU) of 41.26%, Pixel Accuracy of 79.73%, and an Overall Score of 60.50. The inference speed reached 8.3 frames per second. These results indicate the model’s high segmentation accuracy and computational efficiency, providing confidence in the reliability of its semantic segmentation outputs for downstream urban spatial analysis. The resulting panoramas were segmented into 25 primary semantic categories. From these outputs, secondary urban environmental indicators related to the UHI effect were derived and subsequently used as independent variables in the modeling process.

(2) **Building Indicators and POP Processing**

BD was estimated using kernel density analysis based on building centroids. Average BH was calculated from the surrounding neighbourhood, and RD was defined as the total road length per unit area. POP was derived from WorldPop data and processed using ArcGIS Pro to generate a 100-meter gridded distribution [40]. Table 2 summarizes the seven independent variable indicators. BD is defined as the total building footprint area (in square meters) within a 1-hectare grid cell, expressed in units of m^2^/10,000 m^2^. This representation allows for intuitive comparison of built-up intensity across different spatial units.

**Table 2:** Interpretation of data indicators for the independent variables.

GVI	Green View Index	%
SVI	Sky View Index	%
R&PVI	Road and Pavement View Index	%
BD	Building Density	m^2^/10000m^2^
BH	Building Height	m
RD	Road Density	Strip/hm^2^
POP	Population Density	Person/hm^2^

(3) **Remote Sensing LST Data Processing**

The Landsat-9 remote sensing data were processed with atmospheric correction, radiometric correction, and temperature inversion to obtain accurate surface temperature data with a resolution of 30 meters [18,40]. The temperature inversion uses a simplified equation based on Planck’s radiation law [41]:


$$\begin{array}{c} {\text{T}(}^{\circ }\text{C})=\frac{\text{K}\text{2}}{\text{ln}\left(\frac{\text{K}\text{1}}{\text{B}({\text{T}}_{\text{S}})}+\text{1}\right)}-\text{2}\text{7}\text{3}.\text{1}\text{5} \end{array}$$ (1)

where K1 = 774.89 W∙ (m 2 ∙ sr ∙ μm)-1 and K2 = 1321.08 K for Landsat-9 band 10 [18]. To achieve spatial and temporal consistency across all data sources (street view, building data, and LST), this study applied coordinate-based image alignment and Inverse Distance Weighted (IDW) spatial interpolation. Spatial joins ensured all features were matched by geographic coordinates. The IDW algorithm was used to interpolate building and population variables to a consistent analysis unit, ensuring unified spatial resolution and input format for subsequent modeling.

### 3.3 Data collection methods

**Fig 1:**
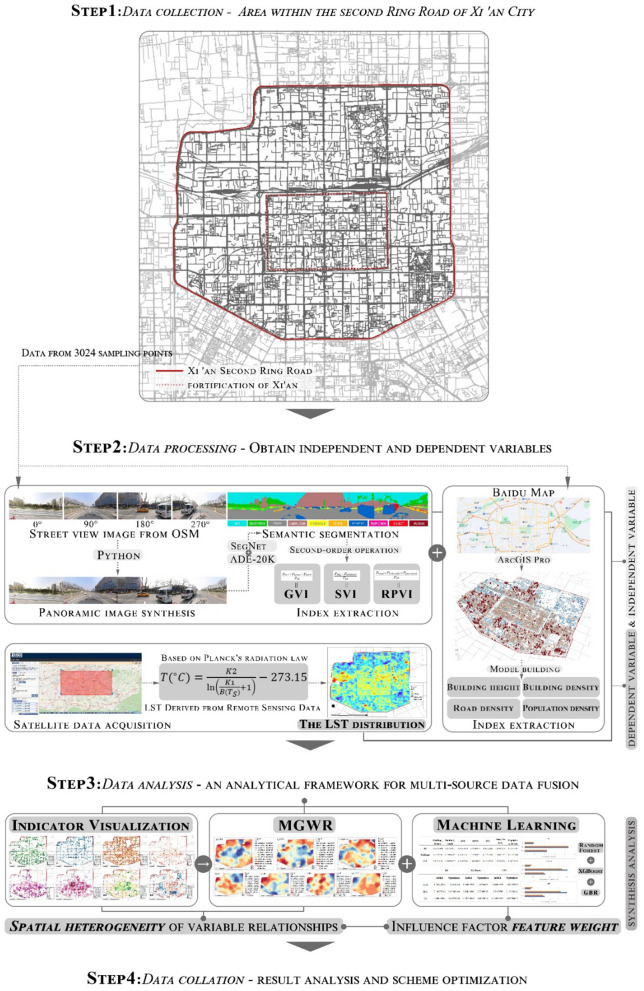
Research areas and technical lines. Base map data © OpenStreetMap contributors, available under Open Database License (ODbL). Analysis and visualization by authors.

The data of this study covers street view images, architectural spatial data, and remote sensing data, all of which are targeted at the area within the second ring road of Xi’an, to ensure that they can comprehensively reflect the characteristics of the urban environment in this area. Fig 1 reveals the scope of the study area and the technical route of the study.

(1) **Street view images data**

Street view images were collected using the Baidu Map API(https://lbsyun.baidu.com/) [20,23], covering all accessible road segments within Xi’an’s second ring road. At each sampling point, images were captured from four directions (0°, 90°, 180°, and 270°) at 25-meter intervals and stitched into panoramic views using a Python-based automation script [39]. A deep learning semantic segmentation model was then applied to extract visual metrics that quantitatively characterize the urban streetscape.

The choice of a 25-meter sampling interval was informed by spatial autocorrelation patterns observed in urban visual features such as green view index (GVI), façade continuity, and sky openness. Prior studies have shown that these features exhibit strong spatial dependency within a range of 30–50 meters in dense urban environments. Accordingly, a 25-meter interval allows for a detailed capture of local morphological variations while maintaining computational efficiency, ensuring the dataset is both spatially sensitive and analytically robust.

(2) **Building spatial data**

The building and road spatial data were obtained from Baidu Map (https://map.baidu.com/) in Shapefile format. The data includes information on the location, area, type, and height of buildings, as well as the length and type of roads [40]. In addition, POP data were downloaded from WorldPop (https://hub.worldpop.org/), which provides gridded population distribution information at a resolution of 100 × 100 meters. Morphological indicators such as BD, BH, and RD can be constructed from these data to provide a basis for analyzing the spatial structure of the city.

(3) **Remote sensing data**

The Landsat-9 satellite was used to obtain the LST data within the second ring of the city of Xi’an [18]. The data were provided by the United States Geological Survey (USGS: https://earthexplorer.usgs.gov/), covering surface temperature information within the second ring of Xi’an City on 23 May 2024, with data cloudiness of 0. After atmospheric correction and radiative transfer modeling, the inversion yielded the LST data at 30 m resolution [12,25].

### 3.4 Data analysis techniques

(1) **Multiscale Geographically Weighted Regression (MGWR)**

The MGWR model further introduces multi-scale analysis based on the traditional GWR model to automatically select the optimal spatial bandwidths for each variable to capture the local regression relationship between the independent variables and the surface temperature more accurately [41,42]. An adaptive bisquare kernel was selected to accommodate local sample variation, and a bandwidth search process based on cross-validation was applied to determine optimal kernel bandwidths for each variable. The multiscale nature of MGWR allows each explanatory variable to operate at a distinct spatial scale, providing more interpretable and accurate estimations of spatial heterogeneity. This bandwidth optimization process for the MGWR model is implemented using the optimization algorithm provided with ArcGIS Pro, which finds the optimal bandwidth based on the AICc criterion, a model selection criterion. This model selection criterion is corrected from the AIC to accommodate smaller sample sizes [43]. The AICc criterion allows us to determine the optimal spatial bandwidth for each variable, thus capturing the local regression relationship between the independent variables and surface temperature more accurately. In this study, the optimal MGWR bandwidth was determined to be 1274.91 m. This bandwidth value balances the fit and complexity of the model and avoids overfitting or underfitting phenomena.

By performing local regression analysis with LST on the visual indicators extracted from street view images, such as BD, BH, and RD, and other independent variables, the MGWR model can reveal the heterogeneity of the influence of factors on the heat island effect in different areas of the city, and generate the distribution map of the regression coefficients at each sampling point, which can provide intuitive support for the causes and spatial characteristics of the heat island effect.

(2) **Machine learning methods**

To further explore the nonlinear relationship and interaction effects between the independent variables and LST, machine learning algorithms such as Random Forest, XGBoost, and Gradient Boosted Regression are used in this paper. By constructing a regression model, the feature importance of each influence factor is automatically identified and ranked. The analysis results of the three types of machine learning are validated against each other, which improves the accuracy of the analysis results and, at the same time, makes the results of the study more refined. The SHAP method is used to provide global and local interpretive analyses of the model prediction results and quantify the contribution of each feature to the LST prediction [44–45] (Table 3).

**Table 3:** Summary of data sources and processing.

Data Type	Source	Resolution	Processing Steps
Street View Images	Baidu Map API	25m interval, 4-directional imagery	Capture → Stitch → Semantic segmentation
Building Spatial Data	Baidu Map + WorldPop	Building footprint 100 × 100m	Shapefile import → Kernel density → Average
Remote Sensing Data	USGS Landsat-9	30 × 30m	Radiometric & atmospheric correction → LST inversion using planck equation

## 4 Results

### 4.1 Results of data processing for the independent variable indicators

**Fig 2:**
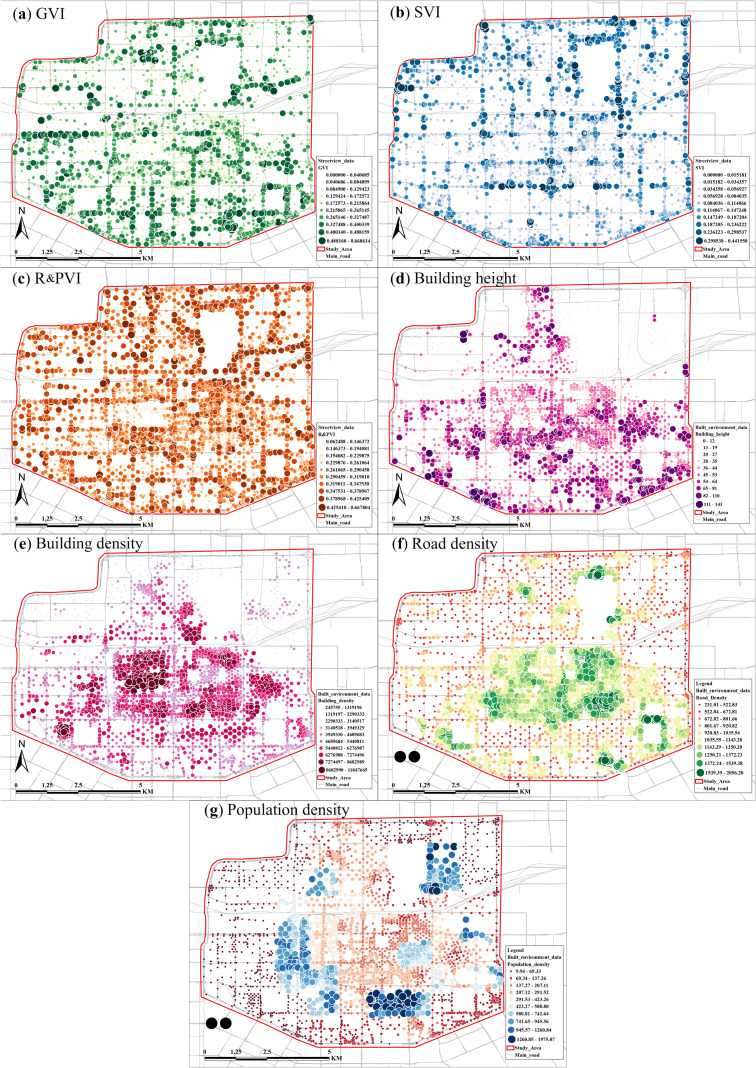
Independent variable processing visualization results. Base map data © OpenStreetMap contributors, available under Open Database License (ODbL). Analysis and visualization by authors.

Fig 2 reveals the distribution of various environmental indicators in the study area within the second ring road of Xi’an. Through the analysis, we can observe the apparent spatial heterogeneity of urban structure and function. BD and RD show significant aggregation effects in the urban core area, where the extreme values of BD mostly reach 868299 m^2^/hm^2^ to 11847665 m^2^/hm^2^, while the RD is mainly located between 1,372 strip/hm^2^ and 1,539 strip/hm^2^.

GVI and R&PVI, on the other hand, were relatively evenly distributed, covering the entire study area. In contrast, the distributions of SVI, BH, and POP show significant regional differences. SVI values are generally higher in the northern region than in the south. BH has significantly higher mean values in the south than in the north, with more high-rise buildings at the southern edge and in the city centre, while BH in the north has the lowest values in many places, between 0 and 12 m.

The distribution of POP shows that POP is higher within the city wall, i.e., the first ring, while it is relatively lower in the periphery of the study area. The highest POP is concentrated in the western and southern parts of the area immediately adjacent to the city wall, and partly in the eastern and northern parts of the study area, mostly clustered around the city wall, with the highest values occurring in the south between about 1260 person/hm^2^ and 1975 person/hm^2^.

### 4.2 Results of data processing for target variables

**Fig 3:**
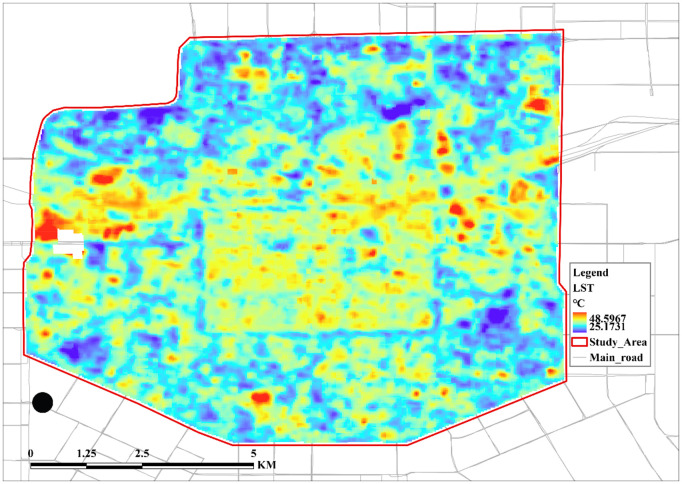
Target variable processing visualization results. Base map data © OpenStreetMap contributors, available under Open Database License (ODbL). Analysis and visualization by authors.

Fig 3 shows the 30-meter resolution surface temperature data obtained after the inversion processing of Landsat-9 remote sensing data. Within the study area, the surface temperature of the urban core and the area near the northern part of the core is significantly higher than that of the low-density area in the periphery of the city, which is characterized by spatial heterogeneity.

LST is a direct quantitative indicator of the UHI effect, reflecting the intensity of heat exchange between the surface and the atmosphere. The spatial distribution of LST and its change pattern can directly reflect the spatial heterogeneity of the UHI effect, and the weights of its influencing factors and the correlation between the two can reveal the spatial distribution law of the UHI effect.

### 4.3 Discriminating the degree of influence of independent variables: Machine learning results reveal the main influences on the heat island effect in complex-built environments

This study used three machine learning models: Random Forest, XGBoost, and gradient-boosted regression to analyse the influence of building information and environmental factors on the LST.

To select the most suitable model, we considered each model’s characteristics: Random Forest shows strong robustness when dealing with high-dimensional data, XGBoost can effectively avoid overfitting by adjusting the learning rate and the depth of the tree, and Gradient Boosted Regression has a strong ability in dealing with non-linear relationships. Before model training, we optimized the hyperparameters of each model by GridSearchCV to ensure the best prediction results.

**Fig 4:**
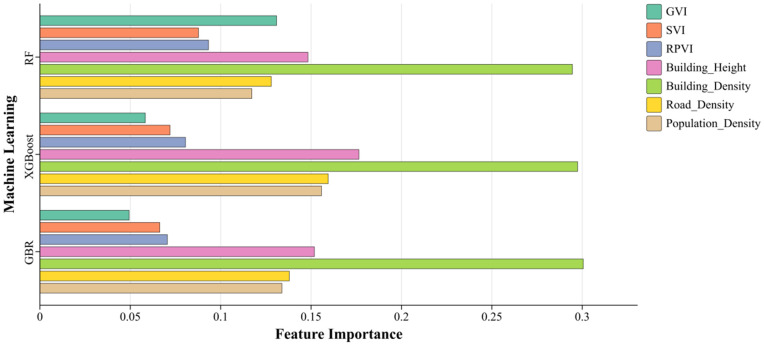
Machine learning feature importance indicator data. Comparison visualisation chart.

**Fig 5:**
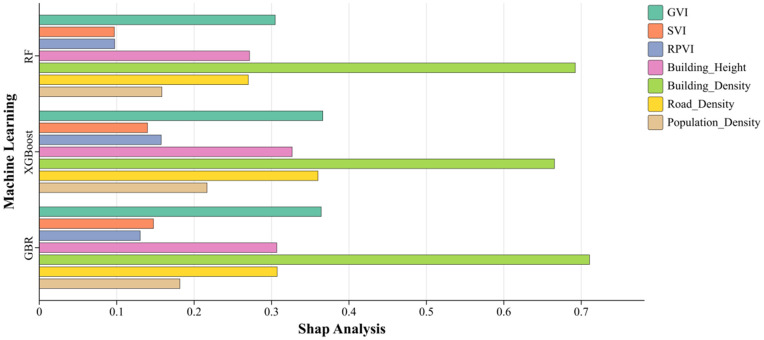
Machine learning SHAP interpretability analysis metrics data. comparison visualisation charts.

Table 4 reveals the results of feature importance and SHAP values of the three machine learning models after analysing the correlation between the data indicators of the independent variable and the target variable, while Figs 4 and 5 visualize both for analysis. Based on the above information, we can comprehensively assess the impact of each feature on LST. The feature importance scores show that BD is essential in all three models. For example, in the XGBoost model, BD has an importance score of 0.297, significantly higher than the other variables, indicating that it has the most significant impact on LST. Areas of high BD lead to an increase in LST due to heat accumulation.

**Table 4:** Mean Absolute SHAP Values and Feature Importance Scores of Independent Variables in Three Machine Learning Models.

	Machine Learning	GVI	SVI	R&PVI	BH	BD	RD	POP
Feature Importance	**Random Forest**	0.130921109	0.087703734	0.093255089	0.148272633	0.29457269	0.12801777	0.117256976
	**XGBoost**	0.058321301	0.071990535	0.080532894	0.176451236	0.29744035	0.159486175	0.155777559
	**Gradient Boosted Regression**	0.049348313	0.066303693	0.070505872	0.151848659	0.300581694	0.137953117	0.13385956
Shap Analysis	**Random Forest**	0.304764113	0.097002335	0.097350479	0.271548105	0.692370348	0.269973458	0.158407797
	**XGBoost**	0.366169631	0.139822006	0.15749079	0.326654315	0.66539526	0.360021979	0.216752604
	**Gradient Boosted Regression**	0.364155203	0.147475824	0.130390838	0.306790173	0.710691154	0.307320386	0.181624025

*SHAP value calculation method: Based on the Shapley value in cooperative game theory, the variation of model prediction is assigned to each feature: firstly, the baseline value (usually defined as the average value of the target variable of the model prediction) is determined by: $y_{base}=\frac{\text{1}}{N}\sum_{i=\text{1}}^{N}\;y_{i}$*, and then, by evaluating the variation of the feature on the model output, we calculate the contribution of each feature to the prediction result of each sample* *−* *that is:* ${SHAPvalue}_{ij}=\phi (x_{ij},f)$*. Finally, using the calculated SHAP values, an explanation is generated for each sample by:* $y_{i}=y_{base}+\sum_{j=\text{1}}^{M}\;{SHAPvalue}_{ij}$*to show how each feature affects the prediction.*

Further SHAP analysis supports this result. The SHAP value of 0.665 for BD in the XGBoost model verifies its significant effect on LST predictions. The SHAP value reflects the contribution of each feature to the prediction outcome for each sample. In this study, we used the mean absolute SHAP values across all samples to assess the global importance of each variable. This approach provides a robust measure of overall feature influence, allowing comparisons across models. The results indicate that BD exerts the strongest influence on surface temperature in dense urban areas, highlighting the roles of the thermal radiation effect and building shadowing [45,46].

Table 5 demonstrates that the prediction accuracy of all three machine learning models is improved through parameter optimization, as evidenced by the reduction of the error metrics MAE and MSE and the stabilization of the R^2^ scores, which suggests that parameter tuning optimization is essential for improving model performance. By evaluating the model performance, the optimized XGBoost model performs the best prediction accuracy, with the MSE decreasing from 2.534 to 2.220 and the coefficient of determination R^2^ increasing from 0.33 to 0.41, which shows a significant performance improvement. In comparison, Random Forest and gradient-boosted regression improve their R^2^ to 0.45 and 0.46, respectively.

**Table 5:** Interpretation of data indicators for the independent variables.

Machine learning	Random Forest		XGBoost		Gradient Boosted Regression	
	Initial	Optimized	Initial	Optimized	Initial	Optimized
**MAE**	1.106442086	1.106610146	1.224692715	1.147364451	1.185489237	1.078393767
**MSE**	2.196375146	2.191888666	2.534090906	2.220589851	2.51059769	2.138320043
R²	0.444090914	0.445226456	0.330208686	0.413070861	0.364560253	0.458784833

While the R^2^ values remain relatively low, this is consistent with previous studies involving urban perception and street-level indicators. The limited explanatory power can be attributed to the complex and nonlinear nature of urban thermal environments, which are influenced by many factors beyond morphological features, such as surface materials, shading conditions, wind flow, and atmospheric conditions. In addition, the use of remotely sensed LST data introduces measurement noise and temporal mismatches that may further affect model performance. Despite this, the observed improvements through tuning and the consistent variable rankings across models confirm the robustness of the proposed framework in identifying key spatial drivers of the UHI effect.

### 4.4 Spatial heterogeneity: The MGWR model reveals regional differences in the effects of different environmental factors on LST

Through local regression analysis, this study reveals significant spatial heterogeneity in the influence of environmental features on LST in different areas within the second ring road of Xi’an. This heterogeneity is not only reflected in the differences in the effects of building form characteristics (e.g., BD and BH) but also the changes in the intensity of the effects of environmental factors (e.g., GVI, RD).

The positive and negative values of the regression coefficients shown in the legend on the right side indicate the positive and negative correlations between the independent variables and the target variables, and the size of the extreme difference of the regression coefficients can indicate the fluctuation amplitude of the regression coefficients between the independent variables and the target variables. the correlation between different independent variables and the target variable reflects different spatial distribution characteristics and fluctuation ranges of regression coefficients. In conjunction with Fig 2, the results of the local regression analyses will be discussed in the following five directions: the core of the study area, the north, the south, the west, and the east.

**Fig 6:**
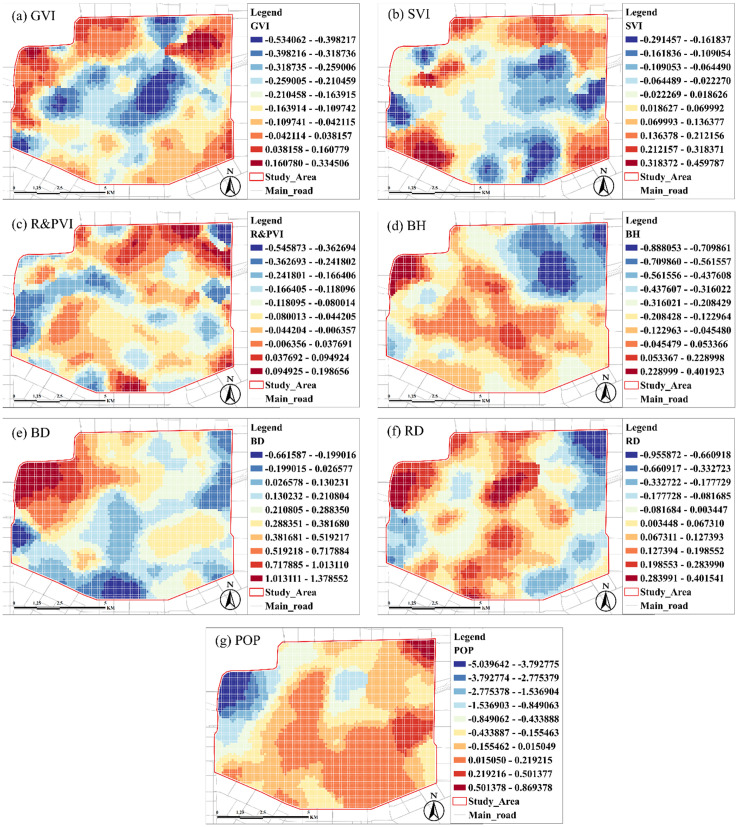
Spatial distribution of the effects of building and environmental factors on surface. Base map data © OpenStreetMap contributors, available under Open Database License (ODbL). Analysis and visualization by authors.

Referring to Fig 2, the core urban area of Xi’an is mainly the old urban area within the city wall, where the scale and density of the old communities are relatively large. In contrast, the POP of the cultural and tourism functional area in its centre is relatively high, with a moderate overall greening. As shown in Figs 6c, f, the GVI ranges from −0.5 to −0.26, higher green coverage results in lower surface temperature in the north side of the old urban area. As shown in Fig 2, the north side of the core urban area is distributed with a complex urban interchanges system and railway stations, and the RD is relatively high, with regression coefficients ranging from 0.13 to 0.40.

The northern area within the second ring road is a high-density urban renewal neighbourhood, especially in dense residential areas with concentrated populations and high building densities. Several regional cultural heritage sites and parks have relatively high green coverage. The regression coefficients for BD range from 0.72 to 1.38, which is overall high and varies widely (Figs 2 and 6a); the areas of cultural heritage sites and parks on the eastern side of the northern region are generally low-built, with regression coefficients for BH ranging from −0.89 to −0.7; and the regression coefficients for the GVI range from −0.53 to −0.32. The north eastern portion of the second ring road has a higher population density, and it is distributed among the neighbourhoods in clusters, with regression coefficients ranging from 0.22 to 1.38. The POP was higher in the north eastern part of the second ring road and clustered with the neighbourhoods, with regression coefficients ranging from 0.22 to 0.87, with more considerable variations (Figs 2 and 6d).

In the southern part of the second ring road, there are sparser low-rise building areas with high green coverage, and in some regions, there are more spacious public green areas and university campuses. The overall BD is low, with an average regression coefficient of about −0.6 (Figs 2 and 6a); the university campus has fewer buildings and high SVI, with regression coefficients ranging from −0.29 to −0.1, which enhances the evacuation of heat from the ground (Figs 2 and 6g).

The western part of the second ring road is dominated by universities and large public green areas, with low BD and abundant greenery. The regression coefficients of their BD range from −0.66 to 0.21, and the overall low building densities effectively suppress the surface temperatures; the higher number of large green spaces in this area leads to lower POP, with regression coefficients ranging from −0.50 to −0.29, which reduces the heat emissions (Figs 2 and 6a); and the high-density high-rise commercial complexes on the south western side increase the sky shading, with the regression coefficients of SVI there ranging from 0.32 to 0.46 (Figs 2 and 6g).

The eastern part of the second ring road is home to university campuses and large heritage parks, with moderate BD and moderate green coverage, making it a typical functional area for education and recreation. A large number of university campuses and heritage parks on its southeast side have moderate BH (Fig 2), which helps to reduce heat accumulation, with regression coefficients ranging from −0.44 to −0.04 for BH and from 0.22 to 0.87 for POP, which, especially in the university area, has a significant increase in the impact on LST at higher POP (Fig 6a, d).

To assess potential multicollinearity among the independent variables, we conducted a Variance Inflation Factor (VIF) analysis. The results show that all VIF values are well below the commonly accepted threshold of 5, indicating no serious multicollinearity problem among the variables. Specifically, the VIF values are as follows: GVI (1.20), SVI (1.58), RPVI (1.27), Building height (1.08), Building density (1.55), Road density (1.11), and Population density (1.03). These results confirm the statistical independence of the explanatory variables, ensuring the robustness of the regression analysis.

**Fig 7:**
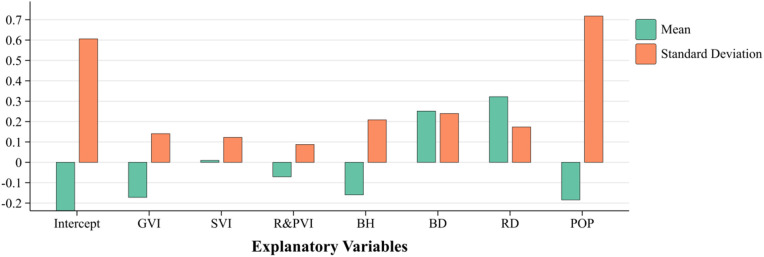
Mean and standard deviation statistics of MGWR regression coefficients.

Overall, the variables showed varying degrees of spatial variability. Table 6 and Fig 6 reveal the mean, standard deviation, extreme, and median values of the regression coefficients of the seven independent variable indicator data after MGWR analysis. Among them, the standard deviation of the regression coefficient of GVI is 0.1408, indicating some differences in the distribution of green space in the study area. Still, the overall significance is relatively low, and the local effect is high (Table 7). Meanwhile, BH (coefficients ranging from −0.8881 to 0.4019) and BD (from −0.6616 to 1.3768) are distributed differently in the core urban area and other areas within the second ring road due to urban planning. Also, RD and POP (coefficients ranging from −5.0396 to 0.8694) show significant spatial heterogeneity (Fig 6). The regression coefficients indicate the influence of key variables on LST. All independent variables show statistical significance at the 99% confidence level (p < 0.01), based on OLS estimation. This reinforces the robustness of the results (Fig 7).

**Table 6:** Summary statistics for MGWR regression coefficient estimation.

Explanatory Variables	Mean	Standard Deviation	Minimum Value	Median	Maximum Value	Robust_Pr
Intercept	−0.2380	0.6061	−2.4361	−0.1525	0.6406	0.000000*
GVI	−0.1721	0.1408	−0.5341	−0.1725	0.3345	0.000000*
SVI	0.0095	0.1225	−0.2915	−0.0131	0.4598	0.000318*
R&PVI	−0.0710	0.0874	−0.5461	−0.0639	0.1987	0.000182*
BH	−0.1596	0.2085	−0.8881	−0.1137	0.4019	0.000000*
BD	0.2514	0.2393	−0.6616	0.2238	1.3768	0.000000*
RD	0.3222	0.1734	−0.9559	0.0536	0.4015	0.013142*
POP	−0.1843	0.7178	−5.0396	−0.0153	0.8694	0.000000*

**Table 7:** Summary of constant and variable coefficients in MGWR model Summary statistics for MGWR regression coefficient estimation.

Explanatory Variables	Intercept	GVI	SVI	R&PVI	BH	BD	RD	POP
Constant	1274.91	1274.91	1274.91	1274.91	1274.91	1274.91	1274.91	1274.91
Correlation	2101	1169	75	95	622	1491	345	512

Finally, through the performance simulation test of the model, Table 8 demonstrates the comparison of the performance indexes after using the two geographic would-be regression weighting models. After comparing the values of specific indicators, it is found that the MGWR model has obvious advantages in fitting effect and spatial heterogeneity capture compared to the traditional GWR model. MGWR can provide more accurate results, especially in the analysis of complex spatial data.

**Table 8:** Summary statistics for MGWR regression coefficient estimation.

Statistics	MGWR	GWR
R²	0.6391	0.6446
Adjusted R²	0.6027	0.5894
AICc	6114.3999	6160.0519
σ²	0.3973	0.4106
Sigma-Squared MLE	0.3609	0.3554
Effective Degrees of Freedom	2747.9894	2618.4722

In summary, among the variables with a slight correlation with LST, the standard deviations of SVI and R&PVI are relatively small, and it is considered that these kinds of factors are more affected by the condition of relatively uniform distribution; among the variables with significant correlation with LST but relatively balanced spatial heterogeneity, the effects of GVI and POP on LST fluctuate relatively little among different regions, and it is considered that they are significantly affected by the synergistic effects of other factors; among the variables with large correlation with LST and equally significant spatial heterogeneity, BD, BH and RD are more prominent.

## 5 Discussion

### 5.1 Spatial heterogeneity analysis of urban building characteristics and land surface temperature

(1) **Influence of BD and BH on LST**

The study shows that BD and BH significantly correlate with LST. In high-density areas, the regression coefficients are all greater than zero, and the fluctuation range is small, indicating that the LST in these areas is more consistently affected by BD and BH. This may be because impervious surfaces in high-density building areas reduce surface albedo, resulting in more solar energy being absorbed and stored; at the same time, the narrow street canyon effect hinders air convection, making it difficult for heat to dissipate effectively. This effect is particularly pronounced in high-density commercial areas, where daytime surface temperature is 2–3°C higher than in surrounding low-density regions.

(2) **Relationship between GVI and LST**

Vegetation reduces LST through shading and evapotranspiration [47]. The regression coefficients of GVI ranged from −0.53 to −0.25 in the high-density area, and vegetation could not only carry away heat by evapotranspiration but also reduce the absorption of surface radiation by shading. This suggests that increasing green cover in high-density areas can effectively mitigate the heat island effect.

(3) **Effect of POP on LST**

POP reflects a significant local effect in the spatial dimension. In the urban centre area, POP showed a positive correlation with LST, probably due to the combined effect of increased BD due to POP, rising traffic flow, and increased energy consumption, which enhanced the heat accumulation effect. However, in the north-western part of the study area, POP and LST showed a significant negative correlation. This may be because although these regions have high population densities, they also have high levels of greenery, and the cooling effect of vegetation counteracts the heat accumulation caused by the increase in POP.

(4) **Spatial heterogeneity**

The effects of BD, BH, RD, GVI, and POP each show distinct spatial effects on LST. In high-density areas, the positive impact of BD and BH on LST is apparent, while the increase of GVI can effectively mitigate this effect. In densely populated areas, LST elevation is driven by BD and shaped by factors like traffic and energy use.

(5) **Interpretation of variables with limited influence**

In addition to the variables showing strong influence, our results also revealed that some indicators—such as SVI and R&PVI—had relatively limited impacts on LST. This can be attributed to their relatively uniform spatial distribution across the study area, which limits their ability to explain spatial differences in surface temperature. Moreover, these variables may exert indirect or context-specific effects that are not fully captured by the MGWR or machine learning models, especially when stronger predictors such as BD and RD dominate the thermal response patterns. In the case of POP, although its overall correlation with LST was moderate, its effect varied across subregions: it was positively associated with LST in central high-density zones but negatively associated in peripheral areas with higher green coverage. These findings highlight the importance of considering both spatial heterogeneity and potential interaction effects when interpreting the role of different urban environmental variables in UHI studies.

### 5.2 Analysis of the interaction mechanism of the urban heat island effect

The study shows that GVI and LST are negatively correlated as a whole. In high-density areas such as urban centres, the geographic regression coefficients of GVI and LST show a core aggregation effect and increase from the core outwards. This suggests that in high-density areas, increasing GVI is having a significant impact on reducing LST. However, in the edge of the study area or low-density areas, although elevating green coverage still has a cooling effect, the effect of greening in reducing LST weakens when influenced by BD, BH, or the structure of green spaces [48].

**Fig 8:**
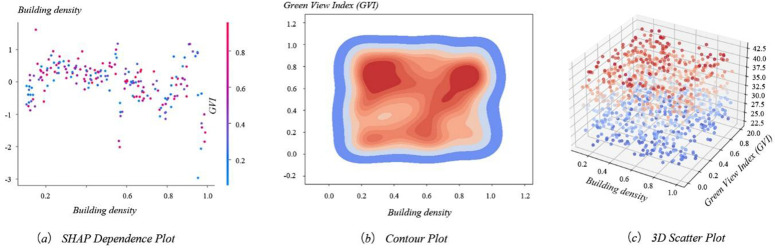
Interaction Between BD, GVI, and LST.

The SHAP dependency plot (Fig 8a) reveals a non-linear and context-dependent relationship between building density (BD) and land surface temperature (LST). When BD is below 0.4, the SHAP value remains relatively low and stable, indicating that building density has little impact on LST in this range. As BD increases from 0.4 to 0.8, LST rises sharply, suggesting that this is a critical threshold zone where impervious surfaces, reduced ventilation, and increased anthropogenic heat begin to significantly amplify the UHI effect.

However, in extremely high-density areas (BD > 0.8), the SHAP values no longer increase monotonically and instead exhibit fluctuations or slight declines. This may result from counteracting effects such as building shadowing, which reduces solar radiation at the surface, or urban canyon effects, which alter heat dissipation through modified air circulation patterns [45–50].

The contour map (Fig 8b) showing the distribution of BD and GVI indicates that GVI is more widely distributed in areas of medium building density (BD = 0.4 to 0.8). However, the distribution of GVI is less in low-density (BD < 0.2) and high-density (BD > 0.8) areas, which correspond to parks, greenfield open spaces, and high-density commercial areas or urban cores in the city, respectively. This implies that the spatial distribution of greenery is more restricted in high-density development areas [49,50].

The effects of BD and GVI on LST were further analysed by a 3D scatter plot (Fig 8c). The results show that areas with high BD and low GVI generally exhibit higher LST. In comparison, regions with low BD and high GVI have significantly lower LST, verifying the critical role of greening in mitigating the heat island effect.

This suggests that high-density development does not necessarily lead to higher LST, and reasonable building layout and greening configuration can still effectively regulate the thermal environment. Therefore, differentiated urban planning strategies should be adopted for different density areas: in medium-density areas, priority should be given to enhancing GVI to reduce LST, while in high-density development areas, simply increasing greening may not be sufficient to minimize temperature effectively, and comprehensive regulation is needed in combination with wind environment optimization, ventilation corridor planning, and other measures. To further examine the potential mediation effect of building density on the relationship between GVI and LST, a structural equation model was constructed. The results show that GVI has no significant effect on building density (p = 1.0), while both GVI (negatively) and building density (positively) significantly affect LST (p < 0.001). These findings suggest that GVI and urban form independently contribute to surface temperature variation, with no evident indirect path from GVI via building density.

### 5.3 Comparison of feature learning methods

When analysing the influencing factors of LST, machine learning methods mainly focus on the overall feature importance, a holistic assessment of the significance of the variables in the whole study area. However, this approach ignores the spatial heterogeneity of the variables and fails to reflect the specific patterns of variable influence on LST within a local area. In contrast, the MGWR method, by calculating the regression coefficients for each geographic location, can reveal the degree of influence of the variables and the trend of change in different spatial regions.

The combined machine learning and MGWR regression analyses found a high degree of consistency in the direction of influence and importance ranking of the main variables. For example, GVI was identified as a relevant factor with a high impact on LST in the machine learning analysis. At the same time, the regression results of MGWR showed that the regression coefficients of GVI ranged from −0.53 to −0.25 in the urban core, indicating its cooling effect. Similarly, BD, BH, and RD all showed up as variables with more significant impacts in the machine learning analyses, and the MGWR results confirmed that their regression coefficients in high-density urban areas were all positive on average and had large values.

Therefore, this study compares and analyses the global feature importance results of machine learning with the local regression coefficients of MGWR, which can effectively achieve mutual validation and thus improve the scientificity and credibility of the research results. This combination of overall and regional analyses verifies the robustness of the research results and lays the foundation for further comprehensive analyses.

To enhance the credibility of the results, we evaluated the robustness of the model outputs and addressed potential sources of uncertainty. First, we compared the outcomes of three machine learning models—Random Forest, XGBoost, and Gradient Boosted Regression. Despite algorithmic differences, all models consistently identified BD, GVI, and RD as the most influential variables, indicating good agreement across methods. Furthermore, performance metrics improved after hyperparameter tuning, with lower MAE and MSE and higher R² values, which demonstrates the stability of the modeling framework. Second, we acknowledge that uncertainties may arise from the input data quality. For example, although LST data were processed with atmospheric correction, they may still be affected by surface complexity and environmental noise. These factors may influence local-scale accuracy but do not alter the overall spatial patterns observed.

### 5.4 Urban planning implications based on spatial heterogeneity

Based on the spatial heterogeneity identified in the MGWR and machine learning models, targeted urban planning strategies can be proposed. In high-density central areas, where LST levels are significantly elevated and GVI remains low, vertical greening, green roofs, and small-scale pocket parks should be prioritized to enhance urban thermal comfort without drastically reducing development intensity. In contrast, peripheral zones with relatively low BD but increasing LST—often due to reduced vegetation continuity or rapid land use changes—should focus on protecting existing green infrastructure and reinforcing ecological corridors. These findings complement previous research on environmental health vulnerabilities in Xi’an’s residential areas suggesting that comprehensive approaches integrating thermal and wind environments are essential for creating healthier urban spaces [51]. Additionally, integrating greening into transport and public spaces can offer dual benefits for both thermal regulation and spatial quality. These region-specific recommendations provide practical guidance for optimizing greening efforts and managing building intensity in future urban development. Our findings align with several recent UHI studies in emphasizing the dominant roles of building density (BD) and urban greenery (GVI) in influencing land surface temperature (LST). For example, Mekhloufi et al. demonstrated in Algiers that free satellite imagery and open‑source tools (e.g., Landsat, QGIS, Google Earth Engine) can effectively map spatial-temporal variations in urban green spaces and their cooling impacts on surface temperature [14]. In addition, a comparative study by Ding et al. in Chengdu and Chongqing, China, revealed that land surface temperature is shaped by a mixture of socio-economic indicators, land-use composition, and 3D building morphology, with each city exhibiting distinct variable importance patterns. While our model confirms these general patterns of greening and morphological influences, it goes further by highlighting localized effects—e.g., road density (RD) plays a more dominant role in Xi’an, likely due to its compact urban structure and transport network. These comparisons support the broader applicability of our modeling framework while underlining the importance of city‑specific morphological contexts in shaping UHI dynamics [22].

## 6 Conclusion

This study combines multi-source data—including remote sensing imagery, building spatial data, and street view images—and utilizes a hybrid model of Multiscale Geographically Weighted Regression (MGWR) and machine learning to investigate the spatial heterogeneity of the UHI effect within Xi’an’s second ring road. The results reveal that Building Density (BD), Green View Index (GVI), and Road Density (RD) are the dominant environmental factors affecting Land Surface Temperature (LST), with marked spatial heterogeneity across different urban zones.

### 6.1 Theoretical implications

(1) **Construction of a multidimensional data fusion framework**

By integrating street view images (extracting visual indicators such as GVI, SVI, etc.), building spatial data (BD, BH, etc.), and remotely sensed LST data, a high-precision matching between street-level 3D morphological features and macro temperature field is achieved. Compared with previous studies, which are primarily based on streetscape and land cover data, this paper further incorporates building height and population distribution, which makes up for the inadequacy of the traditional methods in resolving the vertical thermal environment.

(2) **The proposed MGWR-machine learning integrated analysis method**

Traditional integration methods mainly rely on a single physical or statistical model, which makes it challenging to consider spatial heterogeneity and nonlinear effects. This study resolves the nonlinear interactions between independent and target variables through machine learning models. At the same time, adaptive bandwidth optimization is used to capture the spatial heterogeneity of variables such as BD and GVI using the MGWR model. Through this combined analysis of local and global impacts, we can more comprehensively understand the driving mechanism of the UHI effect, providing a scientific basis for urban planning and environmental management. This approach not only improves the model’s prediction accuracy but also enhances the interpretability of the results, enabling us to formulate more effective mitigation strategies and providing methodological innovations for the refined management of the heat island effect.

### 6.2 Practical implications

The findings of this study yield several practical insights for urban planning and climate resilience.

(1) **Targeted mitigation strategies**

The spatial heterogeneity of BD and GVI suggests that UHI mitigation cannot rely on uniform greening or density control strategies. In high-density cores, greening alone may be insufficient, requiring integrated approaches including wind corridor design, high-albedo materials, and vertical greening. These spatially differentiated strategies are consistent with the concept of proactive environmental strategy, which views environmental responsiveness as a competitive advantage [52]. In contrast, medium-density zones show high responsiveness to increased GVI, making them priority targets for green infrastructure investment.

(2) **Data-driven urban zoning**

The MGWR spatial output provides planners with precise maps of variable impact zones. These can serve as decision-support tools for zoning policies, thermal comfort standards, and environmental equity strategies.

(3) **Scalable and transferable methodology**

The integrated framework is constructed using openly accessible datasets and reproducible modeling tools. The findings and methodology presented in this study offer transferable value to other fast-growing urban contexts that share similar environmental and morphological characteristics, supporting evidence-based strategies in regional or national planning frameworks.

In summary, by integrating multidimensional urban data and advanced spatial analytics, this study not only reveals key driving mechanisms of the UHI effect but also offers scalable and scientifically grounded strategies for mitigating heat stress in dense urban environments.

## 7 Limitations

Although this study achieved valuable results, the following limitations still exist:

(1) The limitations of data accuracy and spatial resolution may lead to some errors in the analysis results in some regions;
(2) This study mainly relies on surface temperature remote sensing data and does not fully incorporate the effects of atmospheric temperature and meteorological factors.
(3) The dynamics of seasonality and climate conditions on the UHI effect have not been thoroughly explored.

Future work will extend the present single-date framework to multi-temporal LST composites (e.g., Landsat time series and ECOSTRESS data) so that seasonal dynamics and model robustness can be examined more comprehensively.

## 8 Future research directions

Future studies may consider integrating higher-resolution data, multiple meteorological indicators, and seasonal dynamic analyses to more comprehensively reveal the formation mechanism of the UHI effect and provide a more accurate theoretical basis and practical guidance for sustainable urban development and environmental management.

Overall, this study provides a new research framework, which provides methodological support for accurately predicting the UHI effect and formulating mitigation strategies through multi-source data fusion and machine learning techniques. The framework deepens the research on the heat island effect. It provides scientific support and practical guidance for sustainable urban development and climate resilience planning in the context of global climate change.

## 9 Nomenclature

**Table:** 

UHI	Urban Heat Island
LST	Land Surface Temperature
MGWR	Multiscale Geographically Weighted Regression
GWR	Geographically Weighted Regression
CFD	Computational Fluid Dynamics

## Supporting information

S1 File S1 Data. fin_data.csv. Original dataset of independent and dependent variables used in the analysis. S2 Data. fin_data.xlsx. Same dataset as S1, provided in Excel format for reproducibility. S3 Data. fin_data_2.csv and fin_data_2.xls. Extended dataset 2, including supplementary indicators for model validation. S4 Data. fin_data_3.csv. Extended dataset 3, providing additional data points for robustness checks. S5 Data. segmentation_result.csv. Semantic segmentation outputs of street view images, including derived indices such as Green View Index (GVI) and Sky View Index (SVI). S6 Text. OLS.pdf. Supplementary results of the Ordinary Least Squares (OLS) regression analysis, including diagnostic plots and summary tables. S7 Data. GWR folder. Geographically Weighted Regression (GWR) model outputs and related spatial results. S8 Data. MGWR folder. Multiscale Geographically Weighted Regression (MGWR) model outputs and coefficient estimation results.
(ZIP)

## References

[1] Li Y-K, C J-P, K G-X. Dynamic and thermodynamic analysis of the urban heat island effect and aerosol concentration. Chinese J Geophys. 2015;58:729–40. doi: 10.6038/cjg20150303

[2] Huo K, Qin R, Zhao J, Ma X. Long-term tracking of urban structure and analysis of its impact on urban heat stress: a case study of Xi’an, China. Ecol Indicators. 2025;174:113418. doi: 10.1016/j.ecolind.2025.113418

[3] Kikon N, Singh P, Singh SK, Vyas A. Assessment of urban heat islands (UHI) of Noida City, India using multi-temporal satellite data. Sustain Cities Soc. 2016;22:19–28. doi: 10.1016/j.scs.2016.01.005

[4] Liu Y, Li Q, Yang L, Mu K, Zhang M, Liu J. Urban heat island effects of various urban morphologies under regional climate conditions. Sci Total Environ. 2020;743:140589. doi: 10.1016/j.scitotenv.2020.140589 32758818

[5] Mirzaei PA, Haghighat F. Approaches to study Urban Heat Island – Abilities and limitations. Build Environ. 2010;45(10):2192–201. doi: 10.1016/j.buildenv.2010.04.001

[6] Oke TR. The energetic basis of the urban heat island. Quart J Royal Meteoro Soc. 1982;108(455):1–24. doi: 10.1002/qj.49710845502

[7] Stewart ID, Oke TR. Local climate zones for urban temperature studies. Bull Am Meteorol Soc. 2012;93(12):1879–900. doi: 10.1175/bams-d-11-00019.1

[8] Arnfield AJ. Two decades of urban climate research: a review of turbulence, exchanges of energy and water, and the urban heat island. Intl J Climatol. 2003;23(1):1–26. doi: 10.1002/joc.859

[9] Voogt JA, Oke TR. Thermal remote sensing of urban climates. Remote Sens Environ. 2003;86(3):370–84. doi: 10.1016/s0034-4257(03)00079-8

[10] Cermak E. Physical modelling of flow and dispersion over complex terrain.

[11] Martilli A, Clappier A, Rotach MW. An urban surface exchange parameterisation for mesoscale models. Boundary Layer Meteorol. 2002;104(2):261–304. doi: 10.1023/a:1016099921195

[12] Tominaga Y, Mochida A, Yoshie R, Kataoka H, Nozu T, Yoshikawa M, et al. AIJ guidelines for practical applications of CFD to pedestrian wind environment around buildings. J Wind Eng Industrial Aerodynamics. 2008;96(10–11):1749–61. doi: 10.1016/j.jweia.2008.02.058

[13] Mekhloufi N, Aquilino M, Baziz A, Richiardi C, Adamo M. Free satellite data and open-source tools for urban green spaces and temperature pattern analysis in Algiers. Int J Appl Earth Observ Geoinform. 2025;139:104482. doi: 10.1016/j.jag.2025.104482

[14] Skamarock WC, Klemp JB, Dudhia J, Gill DO, Barker DM, Wang W. A Description of the advanced research WRF Version 2.

[15] Wei X, Guan F, Zhang X, Van de Weghe N, Huang H. Integrating planar and vertical environmental features for modelling land surface temperature based on street view images and land cover data. Build Environ. 2023;235:110231. doi: 10.1016/j.buildenv.2023.110231

[16] Oliveira A, Lopes A, Niza S. Local climate zones classification method from Copernicus land monitoring service datasets: an ArcGIS-based toolbox. MethodsX. 2020;7:101150. doi: 10.1016/j.mex.2020.101150 33304834 PMC7718173

[17] Liu Y, Lin W, Guo J, Wei Q, Shamseldin AY. The influence of morphological characteristics of green patch on its surrounding thermal environment. Ecol Eng. 2019;140:105594. doi: 10.1016/j.ecoleng.2019.105594

[18] Tasan M, Voosoghi B, Haji-Aghajany S, Amin Khalili M, Di Martire D. InSAR and GNSS data fusion for improved urban heat island estimation using local climate zone classification. Int J Appl Earth Observ Geoinform. 2024;130:103906. doi: 10.1016/j.jag.2024.103906

[19] Yang Q, Xu Y, Chakraborty T, Du M, Hu T, Zhang L, et al. A global urban heat island intensity dataset: Generation, comparison, and analysis. Remote Sens Environ. 2024;312:114343. doi: 10.1016/j.rse.2024.114343

[20] Ahmed RR, Strielkowski W, Štreimikienė D, Salman F, Asim J, Štreimikis J. Enhancing environmental sustainability in Asian textile supply chains: insights from agile practices and mediating variables. JBEM. 2024;25(5):872–91. doi: 10.3846/jbem.2024.21789

[21] Ahmed RR, Kyriakopoulos GL, Streimikiene D, Streimikis J. Drivers of proactive environmental strategies: evidence from the pharmaceutical industry of Asian economies. Sustainability. 2021;13(16):9479. doi: 10.3390/su13169479

[22] Huang X, Wang Y. Investigating the effects of 3D urban morphology on the surface urban heat island effect in urban functional zones by using high-resolution remote sensing data: a case study of Wuhan, Central China. ISPRS J Photogrammetry Remote Sens. 2019;152:119–31. doi: 10.1016/j.isprsjprs.2019.04.010

[23] Chen H, Jeanne Huang J, Li H, Wei Y, Zhu X. Revealing the response of urban heat island effect to water body evaporation from main urban and suburb areas. J Hydrol. 2023;623:129687. doi: 10.1016/j.jhydrol.2023.129687

[24] Guo A, Yang J, Sun W, Xiao X, Xia Cecilia J, Jin C, et al. Impact of urban morphology and landscape characteristics on spatiotemporal heterogeneity of land surface temperature. Sustain Cities Soc. 2020;63:102443. doi: 10.1016/j.scs.2020.102443

[25] Gao W, Liu J, Li S, Xu K, Wang M, Xia Z. Effect of urban morphology on local-scale urban heat island intensity under varying urbanisation: a case study of Wuhan. Sustain Cities Soc. 2025;125:106328. doi: 10.1016/j.scs.2025.106328

[26] Wang J, Lu L, Zhou X, Huang G, Chen Z. Spatio-temporal patterns and drivers of the urban heat island effect in arid and semi-arid regions of Northern China. Remote Sens. 2025;17(8):1339. doi: 10.3390/rs17081339

[27] Hu J, Yang Y, Zhou Y, Zhang T, Ma Z, Meng X. Spatial patterns and temporal variations of footprint and intensity of surface urban heat island in 141 China cities. Sustain Cities and Soc. 2022;77:103585. doi: 10.1016/j.scs.2021.103585

[28] Gao Y, Zhao J, Han L. Exploring the spatial heterogeneity of urban heat island effect and its relationship to block morphology with the geographically weighted regression model. Sustain Cities Soc. 2022;76:103431. doi: 10.1016/j.scs.2021.103431

[29] Shahfahad, Bindajam AA, Naikoo MW, Talukdar S, Asif, Mallick J, et al. Analysing diurnal temperature range and extreme temperature events over Delhi and Mumbai mega cities. Nat Hazards. 2023;120(10):9267–95. doi: 10.1007/s11069-023-06077-9

[30] Zhao L, Oppenheimer M, Zhu Q, Baldwin JW, Ebi KL, Bou-Zeid E, et al. Interactions between urban heat islands and heat waves. Environ Res Lett. 2018;13(3):034003. doi: 10.1088/1748-9326/aa9f73

[31] Zou Z, Yan C, Yu L, Jiang X, Ding J, Qin L, et al. Impacts of land use/ land cover types on interactions between urban heat island effects and heat waves. Build Environ. 2021;204:108138. doi: 10.1016/j.buildenv.2021.108138

[32] Yadav N, Rajendra K, Awasthi A, Singh C, Bhushan B. Systematic exploration of heat wave impact on mortality and urban heat island: a review from 2000 to 2022. Urban Clim. 2023;51:101622. doi: 10.1016/j.uclim.2023.101622

[33] Rao X, Cui H, Dong J, Xiang L, Peng L. Analysis of the surface urban heat island effect and the spatiotemporal heterogeneity of its driving factors. Trans GIS. 2025;29(1). doi: 10.1111/tgis.70013

[34] Wang M, Xu H. The impact of building height on urban thermal environment in summer: a case study of Chinese megacities. PLoS One. 2021;16(4):e0247786. doi: 10.1371/journal.pone.0247786 33887759 PMC8062157

[35] Jordan MI, Mitchell TM. Machine learning: trends, perspectives, and prospects. Science. 2015;349(6245):255–60. doi: 10.1126/science.aaa8415 26185243

[36] Chiang Y-C, Liu H-H, Li D, Ho L-C. Quantification through deep learning of sky view factor and greenery on urban streets during hot and cool seasons. Landscape Urban Plann. 2023;232:104679. doi: 10.1016/j.landurbplan.2022.104679

[37] Huang Z, Duan L, Xu Y, Yang S, Lin Z, Yue H, et al. Exploring the influence of urban green space and urban morphology on urban heat Islands using street view and satellite imagery. Sci Rep. 2025;15(1):23759. doi: 10.1038/s41598-025-07904-8 40610577 PMC12229596

[38] Gao Y, Zhao J, Han L. Quantifying the nonlinear relationship between block morphology and the surrounding thermal environment using random forest method. Sustain Cities Soc. 2023;91:104443. doi: 10.1016/j.scs.2023.104443

[39] Li Z-L, Tang B-H, Wu H, Ren H, Yan G, Wan Z, et al. Satellite-derived land surface temperature: Current status and perspectives. Remote Sens Environ. 2013;131:14–37. doi: 10.1016/j.rse.2012.12.008

[40] Brunsdon C, Fotheringham AS, Charlton ME. Geographically weighted regression: a method for exploring spatial nonstationarity. Geographical Analysis. 1996;28(4):281–98. doi: 10.1111/j.1538-4632.1996.tb00936.x

[41] Fotheringham AS, Charlton ME, Brunsdon C. Geographically weighted regression: a natural evolution of the expansion method for spatial data analysis. Environ Plan A. 1998;30(11):1905–27. doi: 10.1068/a301905

[42] Sugiura N. Further analysis of the data by Akaike’s information criterion and the finite corrections. Commun Statist Theory Methods. 1978;7(1):13–26. doi: 10.1080/03610927808827599

[43] Zhu Y, Zhou H, Hao X. Research progress on urban street canyon heat island effect. In: He B-J, Prasad D, Yan L, Cheshmehzangi A, Pignatta G, eds. Lecture notes in civil engineering. Springer Nature Singapore; 2024: 467–80. doi: 10.1007/978-981-97-8401-1_33

[44] Liu Z, Hu L, Chen H, Li Z, Jiang L. Exploring the combined cooling effect of street canyon geometry and the surrounding built environment. Environ Sci Pollut Res Int. 2024;31(19):28507–24. doi: 10.1007/s11356-024-33012-7 38558341

[45] The cooling effect of urban green spaces in metacities: a case study of Beijing, China’s capital. https://www.mdpi.com/2072-4292/13/22/4601

[46] Wu Z, Zhou Y, Ren Y. Green space-building integration for Urban Heat Island mitigation: Insights from Beijing’s fifth ring road district. Sustain Cities Soc. 2024;116:105917. doi: 10.1016/j.scs.2024.105917

[47] Cui S, Wang X, Yang X, Hu L, Jiang Z, Feng Z. Mapping local climate zones in the urban environment: the optimal combination of data source and classifier. Sensors (Basel). 2022;22(17):6407. doi: 10.3390/s22176407 36080866 PMC9460207

[48] Wang C, Zhang H, Ma Z, Yang H, Jia W. Urban morphology influencing the urban heat island in the high-density city of Xi’an based on the local climate zone. Sustainability. 2024;16(10):3946. doi: 10.3390/su16103946

[49] Wen D, Wang L, Cao Q, Hong M, Wang H, Bian G. A comparative study of the effects of urban morphology on land surface temperature in Chengdu and Chongqing, China. Sci Rep. 2024;14(1):25130. doi: 10.1038/s41598-024-77036-y 39448693 PMC11502834

[50] Ahmed RR, Streimikiene D, Zheng X. The impact of proactive environmental strategy on competitive and sustainable development of organizations. JOC. 2021;13(4):5–24. doi: 10.7441/joc.2021.04.01

[51] Ren J, Wang Y, Liu Q, Liu Y. Numerical study of three ventilation strategies in a prefabricated COVID-19 inpatient ward. Build Environ. 2021;188:107467. doi: 10.1016/j.buildenv.2020.107467 33223598 PMC7669478

[52] Chen J, Ma S, Meng Y, Liu Y, Ren J. Identification and diagnosis of wind health-vulnerable spaces in high-rise residential areas of Xi’an. Buildings. 2025;15(9):1538. doi: 10.3390/buildings15091538

