# Supplementary material for: Analysis of spatial heterogeneity in Xi'an's urban heat island effect using multi-source data fusion
Source: PLoS One. 2025 Oct 17;20(10):e0332885. doi: 10.1371/journal.pone.0332885 (PMC12533849; doi:10.1371/journal.pone.0332885)
Supplement: S1 File — S1 Data. fin_data.csv. Original dataset of independent and dependent variables used in the analysis. S2 Data. fin_data.xlsx. Same dataset as S1, provided in Excel format for reproducibility. S3 Data. fin_data_2.csv and fin_data_2.xls. Extended dataset 2, including supplementary indicators for model validation. S4 Data. fin_data_3.csv. Extended dataset 3, providing additional data points for robustness checks. S5 Data. segmentation_result.csv. Semantic segmentation outputs of street view images, including derived indices such as Green View Index (GVI) and Sky View Index (SVI). S6 Text. OLS.pdf. Supplementary results of the Ordinary Least Squares (OLS) regression analysis, including diagnostic plots and summary tables. S7 Data. GWR folder. Geographically Weighted Regression (GWR) model outputs and related spatial results. S8 Data. MGWR folder. Multiscale Geographically Weighted Regression (MGWR) model outputs and coefficient estimation results. (ZIP) [file pone.0332885.s001.zip › S6_Text.pdf]

OLS 结果汇总 - 模型变量

变量	系数 [a]	标准差	t 统计量	概率 [b]	Robust_SE	Robust_t	Robust_Pr [b]	VIF [c]
截距	34.096776	0.196153	173.827590	0.000000*	0.207521	164.305461	0.000000*	--------
GVI	-3.412457	0.257983	-13.227424	0.000000*	0.258176	-13.217544	0.000000*	1.175150
SVI	1.781805	0.492246	3.619744	0.000314*	0.492777	3.615841	0.000318*	1.566546
RPVI	-1.756256	0.474550	-3.700888	0.000231*	0.466662	-3.763439	0.000182*	1.262187
BUILDING_HEI	-0.012282	0.001338	-9.176078	0.000000*	0.001303	-9.423836	0.000000*	1.130799
BUILDING_DEN	0.000000	0.000000	23.556493	0.000000*	0.000000	22.589160	0.000000*	1.452692
HEIGHT_WIDTH	-0.007111	0.004798	-1.482078	0.138437	0.002866	-2.481008	0.013142*	1.097595

OLS 诊断

输入要素	OLS	因变量	LST
观测值个数	3097	阿凯克信息准则(AICc)['d']	11927.215563
R 平方的倍数['d']	0.273255	校正 R 平方['d']	0.271844
联合 F 统计量['e']	193.639530	Prob(>F), (6,3090)自由度	0.000000*
联合卡方统计量['e']	1397.125862	Prob(>卡方), (6)自由度	0.000000*
Koenker (BP)统计量['f']	96.360722	Prob(>卡方), (6)自由度	0.000000*
Jarque-Bera 统计量['g']	8.852245	Prob(>卡方), (2)自由度	0.011961*

解释注意事项

* 数字旁的星号表示在统计学上具有显著性的 p 值(p < 0.01)。

[a] 系数: 表示每个解释变量与因变量之间的关系的强度和类型。

[b] 概率和稳健概率(Robust_Pr): 星号(*)表示系数具有统计学上的显著性(p < 0.01); 如果 Koenker (BP)统计量 [f]

具有统计学上的显著性，则使用稳健概率列(Robust_Pr)来确定系数显著性。

[c] 方差膨胀因子(VIF): 较大的方差膨胀因子(VIF)值(> 7.5)表明解释变量存在冗余。

[d] R 平方和阿凯克信息准则(AICc): 模型拟合度/性能的测量。

[e] 联合 F 统计量和卡方统计量: 星号(*)表示整个模型的显著性(p < 0.01); 如果 Koenker (BP)统计量 [f]

具有统计学上的显著性，则使用卡方统计量来确定整个模型的显著性。

[f] Koenker (BP)统计量: 当此测试具有统计学上的显著性时(p <

0.01)，表示建模的关系不一致(由于不稳定性或异方差导致)。您应该依据稳健概率(Robust_Pr)来确定系数显著性以及依据卡方统计量来确定整个模型的显著性。

[g] Jarque-Bera 统计量: 当此测试具有统计学上的显著性时(p < 0.01)，表示模型预测是片面的(残差未正态分布)。

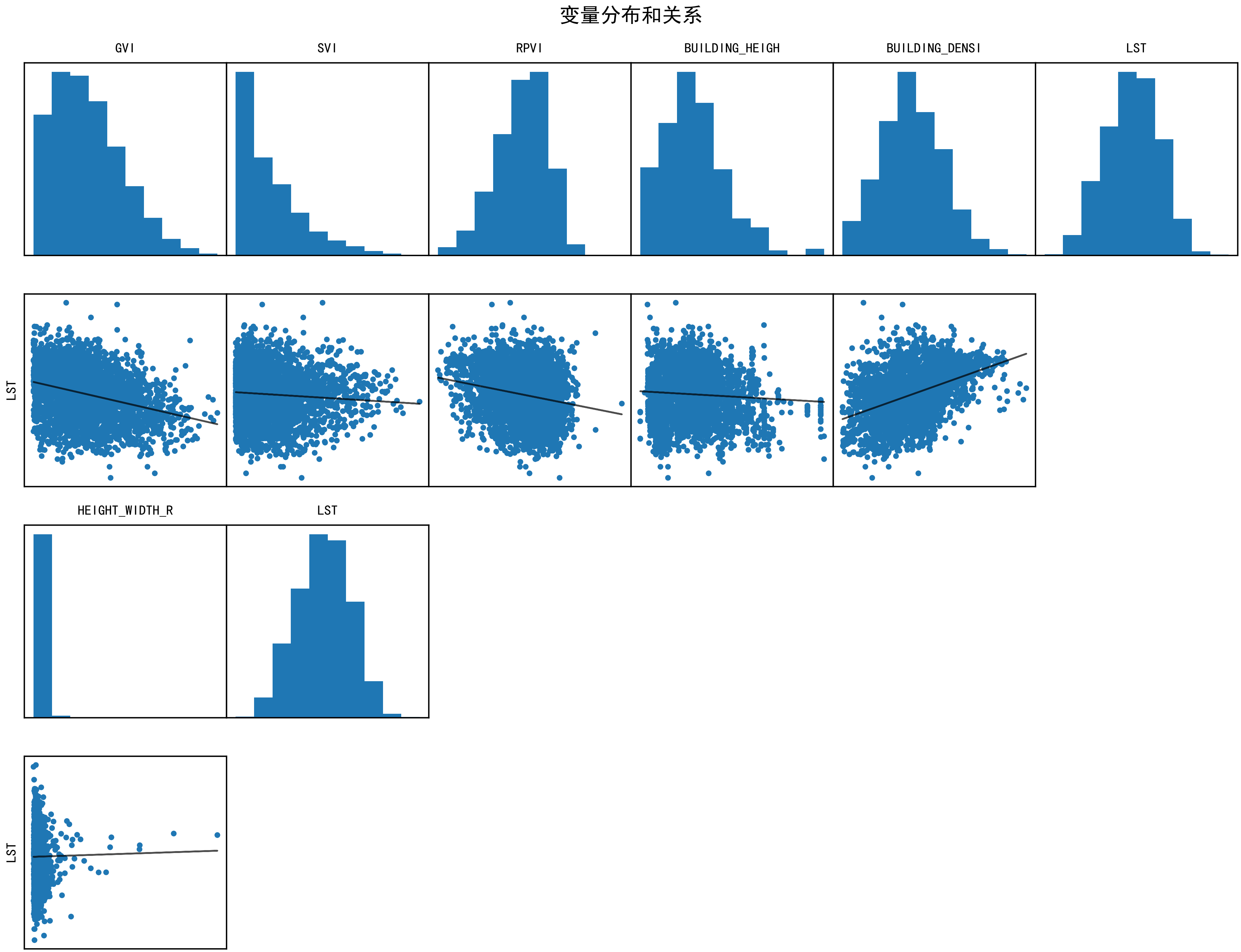
变量分布和关系
GVI
SVI
RPVI
BUILDING_HEIGH
BUILDING_DENSI
LST
LST
HEIGHT_WIDTH_R
LST
LST

变量分布和关系（续）

以上显示的是每个解释变量和因变量的直方图和散点图。直方图显示了每个变量的分布方式。OLS 并不要求变量呈正态分布。尽管如此，如果您难以查找对应的模型，则可尝试对偏态分布的变量进行变换以查看是否可以获得更好的结果。

散点图描述了每个解释变量和因变量之间的关系。较强的关系将显示为对角线，而且倾斜方向会指示关系为正还是为负。如果发现了任何非线性关系，请尝试转换变量。有关详细信息，请参阅“回归分析基础知识”文档。

标准化残差的直方图

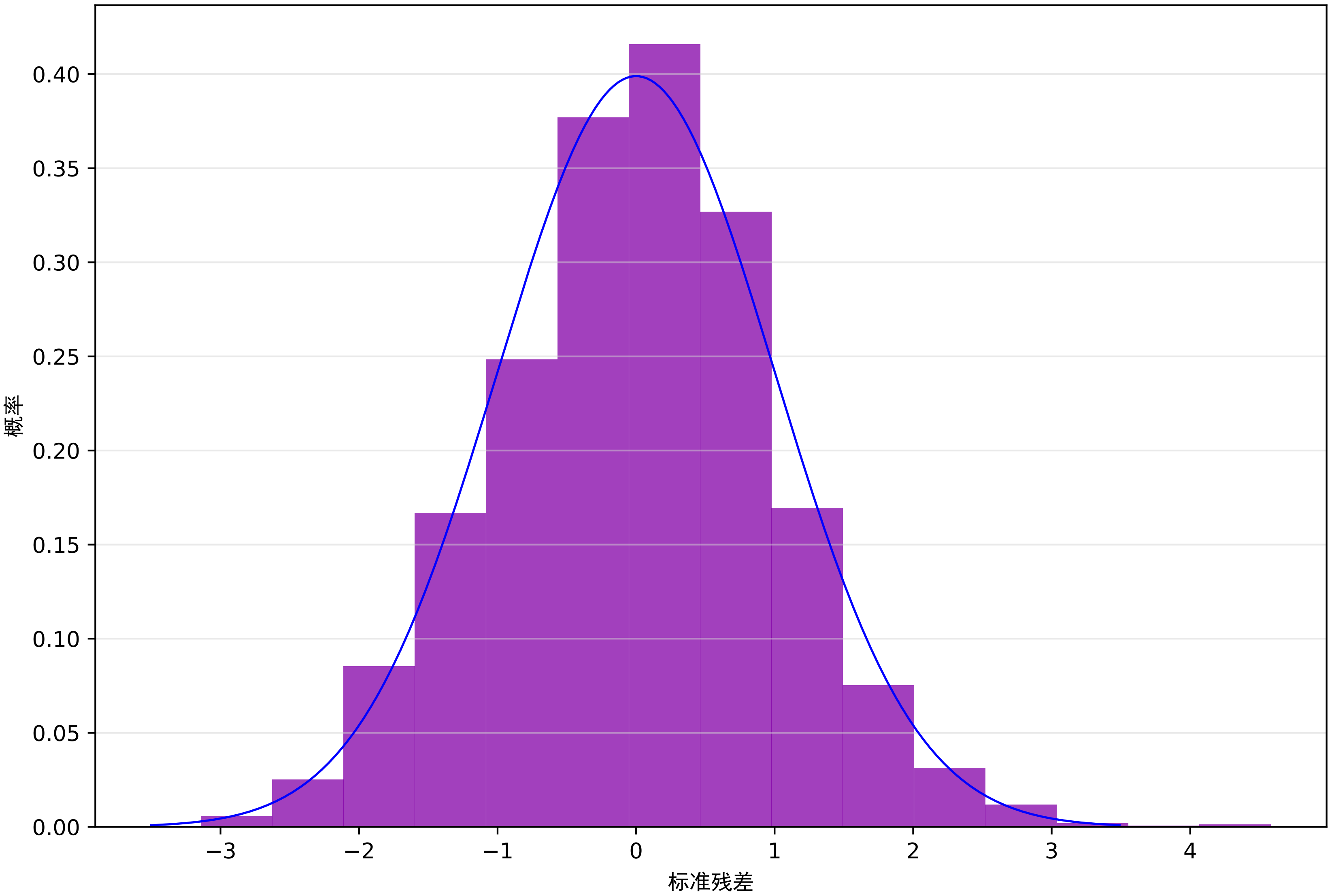

理想情况是残差的直方图与正态曲线相匹配，如上面的蓝色所示。如果直方图与正态曲线之间存在明显差异，则您的模型可能有偏差。如果偏差严重，则模型还可以由具有[illegible]
显著性的 Jarque-Bera p 值(*)来表示。

残差与预测图

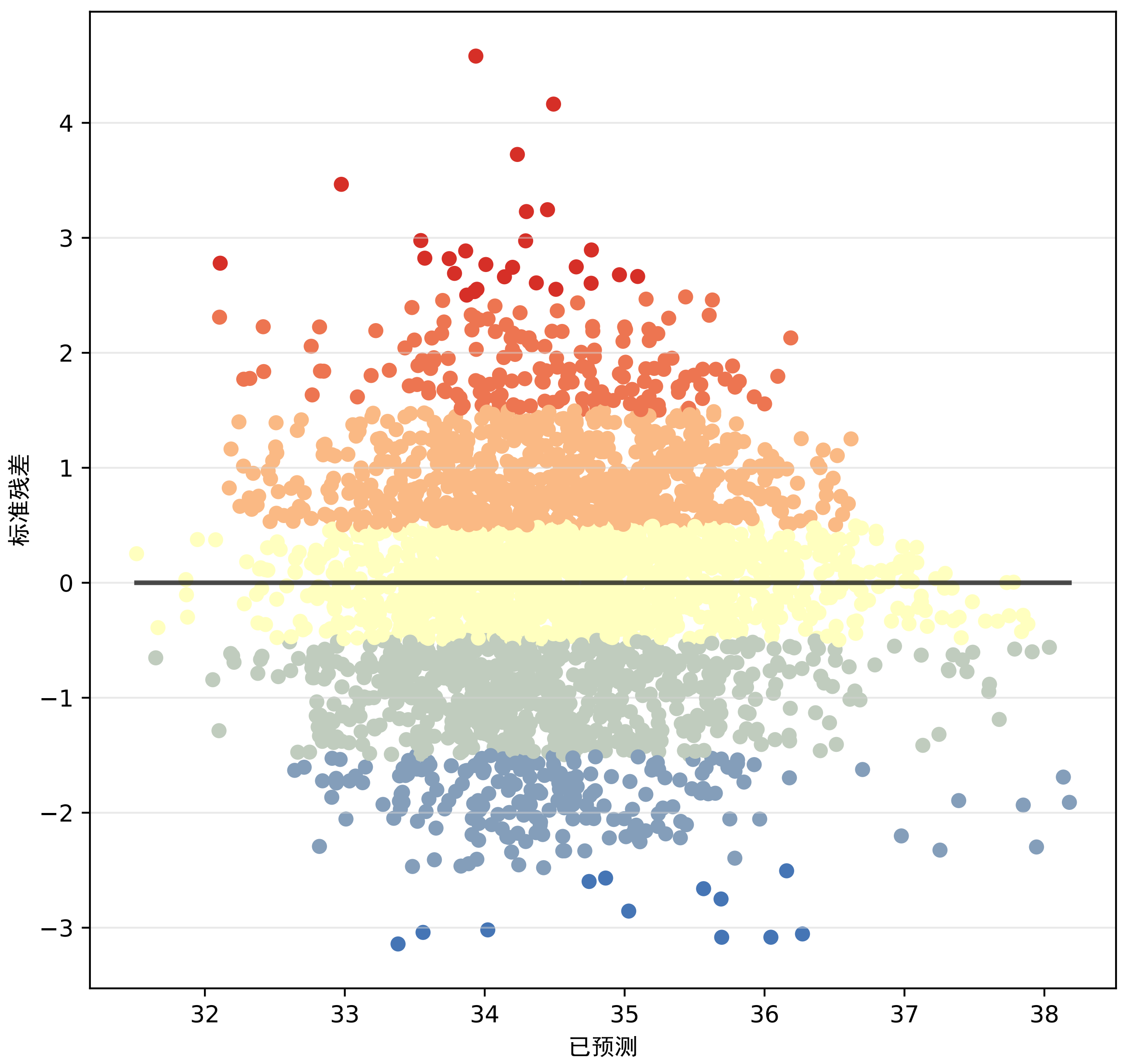

此为与预测的因变量值相关的残差图(位于预测值之上或之下的模型)。对于一个正确指定的模型，该分散图几乎不具有结构，呈随机状(参见右图)。如果该图具有结构，则结构的类型可能是帮助您找出下一步发展的重要线索。

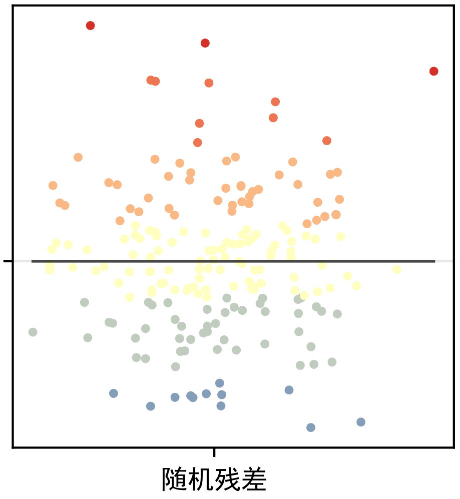

普通最小二乘法参数

参数名称	输入值
输入要素	OLS
唯一 ID 字段	id
输出要素类	
因变量	LST
解释变量	GVI
	SVI
	RPVI
	BUILDING_HEIGHT
	BUILDING_DENSITY
	HEIGHT_WIDTH_RATIO
选择集	False
